# Interactions of metals and Apolipoprotein E in Alzheimer’s disease

**DOI:** 10.3389/fnagi.2014.00121

**Published:** 2014-06-12

**Authors:** He Xu, David I. Finkelstein, Paul A. Adlard

**Affiliations:** The Florey Institute of Neuroscience and Mental Health, The University of MelbourneMelbourne, VIC, Australia

**Keywords:** Apolipoprotein E, Alzheimer’s disease, zinc, copper, metals

## Abstract

Alzheimer’s disease (AD) is the most common form of dementia, which is characterized by the neuropathological accumulation of extracellular amyloid plaques and intracellular neurofibrillary tangles (NFTs). Clinically, patients will endure a gradual erosion of memory and other higher order cognitive functions. Whilst the underlying etiology of the disease remains to be definitively identified, a body of work has developed over the last two decades demonstrating that AD plasma/serum and brain are characterized by a dyshomeostasis in a number of metal ions. Furthermore, these metals (such as zinc, copper and iron) play roles in the regulation of the levels of AD-related proteins, including the amyloid precursor protein (APP) and tau. It is becoming apparent that metals also interact with other proteins, including apolipoprotein E (ApoE). The Apolipoprotein E gene (APOE) is critically associated with AD, with APOE4 representing the strongest genetic risk factor for the development of late-onset AD. In this review we will summarize the evidence supporting a role for metals in the function of ApoE and its consequent role in the pathogenesis of AD.

## Introduction

Apolipoprotein E (ApoE) is the predominant apolipoprotein in the brain where it is synthesized and secreted primarily by astrocytes in high-density lipoprotein (HDL)-like particle (Bu, 2009). A primary function of ApoE is to serve as a ligand for the low-density lipoprotein (LDL) receptor family of proteins, which mediate delivery of cholesterol to neurons. That function is essential for axonal growth, synaptic formation and remodeling and all of those events are important for learning, memory formation and neuronal repair (Mauch et al., 2001; Pfrieger, 2003). Decreases in the levels of ApoE or LDL receptors lead to synaptic remodeling impairment and a progressive loss of synapses in the cortex and hippocampus (Mulder et al., 2004; Liu et al., 2010).

ApoE is also a polymorphic protein with three common allele variants: APOE2, APOE3 and APOE4. The APOE4 gene is the strongest and only confirmed genetic risk factor for the development of late onset Alzheimer’s disease (LOAD), which enhances the risk level by three times in heterozygous individuals and by twelve times in homozygous individuals (Bertram, 2009). The least frequent APOE2 allele (found in 5–10% of individuals) seems to have a protective effect against the development of AD while the most frequent APOE3 allele (found in 70–80% of the population) represents intermediate risk (Corder et al., 1994; Mahley and Huang, 2006). The structural differences between the three ApoE isoforms is limited to amino acid residues 112 and 158, where either cysteine or arginine is present: ApoE2 (Cys112, Cys158), ApoE3 (Cys112, Arg158) and ApoE4 (Arg112, Arg158; Mahley and Rall, 2000). The single amino acid difference at these two positions affects the structure of ApoE isoforms and their ability to bind lipids, receptors and amyloid beta (Aβ), the latter which is the main constituent of the extracellular plaques found in the AD brain (Zhong and Weisgraber, 2009; Chen et al., 2011; Frieden and Garai, 2012).

The connection between metals, Aβ and abnormal forms of tau (as found in the neurofibrillary tangles (NFT) present in the AD brain) has been investigated extensively in the pathogenesis of AD (Grasso et al., 2012; Greenough et al., 2013; Wärmländer et al., 2013). However, the effects of metallation on ApoE are less well known. In this review, evidence supporting the hypothesis that zinc and copper play a role in the function of ApoE will be covered, along with the key points on the current understanding of the influence of ApoE and metals on the pathogenesis of AD.

## ApoE and its role in AD pathogenesis

APOE4 has been found to be associated with an increased prevalence of AD and a lower age of onset. Clinical data shows the frequency of AD and mean onset age are 91% and 68 years old in APOE4 homozygous carriers, 47% and 76 years old in APOE4 heterozygous carriers, and 20% and 84 years old in APOE4 non-carriers (Corder et al., 1993; Rebeck et al., 1993); suggesting that APOE4 genotype confers a significantly higher risk of development of AD with an earlier age of onset in a gene dose-dependent manner. It has also been reported that the prevalence of an E4 allele is considerably higher in mild cognitive impairment (MCI) than in control individuals (Pa et al., 2009); with APOE4 MCI individuals showing poorer memory performance at an earlier stage in AD compared with non-carriers (Smith et al., 1998). APOE4 can also influence cognition in healthy people. Healthy APOE4 carriers show an accelerated longitudinal decline in memory tests (Caselli et al., 2004, 2007). In the brain, ApoE mediates delivery of cholesterol to neurons, which is essential for axonal growth, synaptic formation and remodeling and all of those events are important for learning, memory formation and neuronal repair (Mauch et al., 2001; Pfrieger, 2003). Astrocytes preferentially degrade ApoE4, leading to reduced ApoE4 secretion and ultimately to reduced brain ApoE levels (Riddell et al., 2008). Taken together therefore, the lack of functional ApoE present in AD is likely to directly contribute to the cognitive impairment seen in this disease.

One of the first pieces of evidence linking ApoE to AD pathology was the observation of ApoE immunoreactivity in extracellular amyloid plaques and NFTs (Namba et al., 1991). It has since been shown that ApoE forms complexes with Aβ and these complexes are thought to influence Aβ deposition and clearance (Wildsmith et al., 2013). Aβ deposition detected by Pittsburgh Compound B positron emission tomography (PIB-PET) follows a strong APOE allele-dependent pattern (E4 > E3 > E2) (Kok et al., 2009; Morris et al., 2010; Castellano et al., 2011). An ApoE isoform-specific effect on the amount of Aβ accumulation as well as in the number of amyloid plaques was also found in amyloid precursor protein (APP) transgenic mice expressing different human ApoE isoforms (E4 > E3 > E2; Fagan et al., 2000, 2002; Fryer et al., 2005b). The mechanisms underlying isoform-specific influences on Aβ aggregation and accumulation in the brains are not fully understood, but it’s likely due to their different abilities to clear Aβ (Wildsmith et al., 2013). *In vitro* and *in vivo* studies show that many ApoE receptors are involved in ApoE-mediated Aβ clearance from the brain (Bu, 2009; Kim et al., 2009; Holtzman et al., 2012). A recent study demonstrated that ApoE loses its ability to clear Aβ when ApoE is cleaved at the hinge region of ApoE (Jones et al., 2011). After cleavage, the ApoE-Aβ complex cannot bind to ApoE receptors due to the lack of N-terminal ApoE which contains the binding sites of ApoE receptors. It also has been demonstrated that ApoE4 is more susceptible to the cleavage at hinge region (Jones et al., 2011), increasing the likelihood of the ApoE receptor binding region (N-terminal ApoE) being cleaved, which supports the idea that ApoE4 has the least ability to clear Aβ compared to ApoE2 and ApoE3. Another supportive finding is that ApoE4 clears Aβ at blood brain barrier (BBB) via the very low-density lipoprotein receptor (VLDLR) whereas Aβ is cleared at a higher rate in the presence of ApoE2 and ApoE3 by both VLDLR and lipoprotein related protein 1 (LRP1; Deane and Zlokovic, 2007).

It has also been proposed that the poor stability, clearance and poor lipidation status of ApoE4 accounts for its contribution to an elevated risk for the development of AD. ApoE4 is the least stable of all three ApoE isoforms (Morrow et al., 2002) and has been reported to be preferentially susceptible to proteolytic degradation into cytotoxic fragments (Huang et al., 2001). Much higher levels of ApoE fragments are detected in the brains of AD patients (Huang et al., 2001; Harris et al., 2003; Jones et al., 2011) and these fragments have been shown to damage hippocampal neurons and result in memory impairment (Harris et al., 2003; Andrews-Zwilling et al., 2010).

ApoE4 is a less effective lipid carrier under physiological conditions than ApoE3 or ApoE2 (Michikawa et al., 2000; Hara et al., 2003). Lipidation of ApoE is mediated primarily by ATP-binding cassette A1 (ABCA1) and the lipidation status of ApoE is related to its Aβ-binding properties (Tokuda et al., 2000). Reducing ApoE lipidation status by ablating ABCA1 in APP transgenic mice markedly enhances brain amyloid plaque levels, and conversely, enhancing ApoE lipidation status by the up-regulation of ABCA1 significantly reduced amyloid load (Kim et al., 2009). These results are consistent with the hypothesis that non-lipidated ApoE in the brain can stimulate Aβ aggregation and deposition (Hatters et al., 2006) while lipidated ApoE facilitates the clearance of Aβ and it is much less susceptible to proteolysis than lipid-free ApoE (Weisgraber et al., 1994; Narayanaswami et al., 2004). Some ApoE receptors and ABCA1 appear to influence ApoE expression and lipidation (Hirsch-Reinshagen et al., 2004; Wahrle et al., 2004; Fryer et al., 2005a; Liu et al., 2007; Wahrle et al., 2008).

Based on the pathological definition of the disease, AD is associated not only with the abnormal accumulation of amyloid plaques, but also with the accumulation of NFTs which form intracellularly and are composed primarily of aggregated phosphorylated and acetylated tau (Iqbal et al., 2010). Tau primarily stabilizes microtubules, and its aggregation in AD causes deficits through a loss-of-function mechanism. Recently, evidence has also shown that when it is abnormally modified, tau becomes enriched in dendritic spines where it can interfere with neurotransmission (Morris et al., 2011). Evidence from *in vivo* and *in vitro* studies indicates that ApoE3 and ApoE4 function differently with respect to the phosphorylation and aggregation of tau. ApoE3 binds to the microtubule-binding repeat regions of tau with its N-terminal domain (Strittmatter et al., 1994), however, this interaction can be impeded by the phosphorylation of tau. On the other hand, whilst ApoE4 has been shown to not significantly interact with tau (Strittmatter et al., 1994), it does increase tau phosphorylation and accumulation in the neuronal soma and dendrites, facilitating the formation of NFTs during aging and AD progression (Harris et al., 2003; Brecht et al., 2004; Andrews-Zwilling et al., 2010). One proposed mechanism is that ApoE4 can activate the extracellular signal-regulated kinases (ERK) pathway in AD brains and lead to tau phosphorylation, which is likely modulated by zinc (Harris et al., 2004).

## The involvement of metals in AD pathogenesis

The formation of the classical neuropathological features of AD are not only influenced by APOE genotype, but also mediated or triggered by an imbalance of metal ions. Altered metal homeostasis has been demonstrated in the brain and plasma/serum in AD patients. Compared with age-matched control, AD patients show elevated zinc and copper in cerebrospinal fluid (CSF; Hozumi et al., 2011), whereas plasma and serum zinc was found to be lower (Vural et al., 2010). Free copper in the blood of AD patients is substantially higher than controls (Squitti et al., 2014). In addition, the concentration of zinc, copper, and iron in brain parenchyma (350 µM, 70µM, and 340 µM, respectively) are further elevated in AD patients (800 µM, 300 µM, and 700 µM, respectively; Lovell et al., 1998). These metals are also enriched in both senile plaques and NFTs (Ayton et al., 2013). Zinc, along with copper and iron (released during neural transmission), directly bind to Aβ and accelerate its aggregation and accumulation into amyloid plaques (Morante, 2008; Altamura and Muckenthaler, 2009). Therefore, the zinc, copper and iron sequestration into amyloid deposits is thought to result in a loss of cellular and synaptic metals. The loss of synaptic zinc is particularly relevant to the maintenance of normal cognition. An important regulator of synaptic zinc is the zinc transporter-3 (ZnT3) protein which is essential for loading zinc into synaptic vesicles (Linkous et al., 2008). It has been shown that ZnT3 levels decrease with aging in the brains of both mice and humans and are reduced even further in the brains of AD patients (Adlard et al., 2010). ZnT3 KO mice display defects in learning and memory at 6 months of age, and the authors suggest that these mice provide a phenocopy for the synaptic and memory deficits of AD (Adlard et al., 2010). In addition, copper is another important metal involved in the cognitive decline in AD. Free copper in blood is potentially toxic, particularly if the free copper pool expands, as it does in Wilson’s disease (Brewer et al., 1998). More importantly, there’s a strong positive correlation between the level of free copper and the severity of cognitive loss in AD (Squitti et al., 2006), which can be observed over a given period of time (Squitti et al., 2009).

Although we still do not know if the metal ion dyshomeostasis present in AD is a cause or consequence of the disease, there is a growing body of evidence showing a direct correlation between metal ions and key AD-related key proteins. Both zinc and copper facilitate Aβ aggregation. Aβ tends to form fibrils in the presence of zinc, whereas in the presence of copper it prefers to form oligomers (Tõugu et al., 2009). The copper-Aβ oligomer complex has been shown to be more toxic than the zinc Aβ fibrils, which in some conditions actually confer protection (Rosenblum, 2014). Metal dyshomeostasis is also involved in the regulation of other AD-related proteins, like APP and tau. Zinc, for example, regulates the activity of some of the secretases involved in the processing and function of APP, with α-secretase activity up-regulated by zinc indirectly through a disintegrin and metalloproteinase (ADAM; Lammich et al., 1999); however, the activity of the γ-secretase complex is inhibited by zinc (Hoke et al., 2005). The copper binding domain of APP (histidine residues 149 and 151) is crucial for APP stability and metabolism (Spoerri et al., 2012) and copper enhances APP dimerization and promotes Aβ production (Noda et al., 2013). Consistent with this concept, APP knockout mice have elevated copper levels in the cerebral corte (White et al., 1999). These studies show that APP may directly influence copper homeostasis, and its interactions with copper may be also neurotoxic.

Metals are also involved in tau pathology, and are enriched in tangle-bearing neurons (Sayre et al., 2000). Synaptically released zinc induces tau hyper-phosphorylation through pathways including Src-dependent, glycogen synthase kinase 3β (GSK3β) and ERK pathways (Lei et al., 2011; Xiong et al., 2013). Copper directly binds to tau (Martic et al., 2013) and regulates its aggregation and phosphorylation (Squitti et al., 2006; Zhou et al., 2007). Aberrant activation of cyclin-dependent kinase 5 (CDK5) was found to be correlated with the tau pathology after chronic copper exposure in a mouse model of AD (Kitazawa et al., 2009). Iron binding to the hyper-phosphorylated tau protein also facilitates the formation of the NFTs (Altamura and Muckenthaler, 2009) and the iron chelator, deferoxamine (DFO), decreases iron-induced activities of CDK5 and GSK3β and tau phosphorylation (Guo et al., 2013). Thus, the development of the two most prominent pathological features of the AD brain, plaques and tangles, are likely to be mediated by metal ions. This area has been extensively reviewed in the past (Adlard and Bush, 2006; Bush and Tanzi, 2008; Duce and Bush, 2010; Hung et al., 2010; Ayton et al., 2013), and supports the notion of the regulation of metal homeostasis as a promising area of investigation for future AD therapeutics.

## Evidence supporting the link between metals and ApoE

The mechanism by which ApoE4 is associated with AD is still unknown; however, an emerging linkage between metals and ApoE might give a clue. Evidence shows that ApoE isoforms bind to metals such as zinc, copper and iron (that are also involved in the pathogenesis of AD), with the affinity for copper being greater than for iron and zinc (Miyata and Smith, 1996). The precise binding sites for metals on ApoE have yet to be determined, but the four-helix bundle of the N-terminus may allow a coordination of metals (Miyata and Smith, 1996). The metal sequestration properties of ApoE might present metals to Aβ peptides, leading to amyloid deposition or it might account for the antioxidant function of ApoE in AD development. Furthermore, studies support the notion that ApoE2 has the highest affinity for zinc and ApoE4 has the lowest. This is likely a result of structural differences amongst the three isoforms. Cysteine is a strong ligand for zinc, arginine is not (Karlin and Zhu, 1997), so the affinity for zinc is predicted to be greatest for ApoE2 which has cysteine residues at amino acid position 112 and 158 and weakest for ApoE4 which lacks cysteine residues. This likelihood is supported by the results showing that ApoE protects Aβ from zinc-induced precipitation in the order of ApoE2 > ApoE3 > ApoE4 (Moir et al., 1999). It is also speculated that ApoE4 has a reduced copper binding capacity because of its lack of cysteine residues (Hung et al., 2013). Although direct evidence for the metal:ApoE interaction needs to be demonstrated, these data clearly provide a potentially important avenue of investigation for understanding the mechanism underlying the higher risk of AD in APOE4 carriers. Metal ions, such as zinc, play an essential role in stabilizing protein structures and contributing to protein function (Wang et al., 2010). We further speculate that metal binding might help to stabilize ApoE in an order of E2 > E3 > E4 in the proteolytic process, which leads to less ApoE4 and more ApoE4 fragments. This is consistent with the previous findings that APOE4 carriers have less full-length ApoE but more ApoE fragments in brain parenchyma and plasma than APOE2 carriers (Riddell et al., 2008; Gupta et al., 2011); with decreased ApoE levels in APOE4 carriers considered an important factor for AD onset/development (Verghese et al., 2011; Holtzman et al., 2012). Therefore, the stability of ApoE may be affected by metals, and this may help account for the differential effect of the three ApoE alleles on the development of AD.

There are also a number of studies that have investigated the effect of metals on the expression levels of apolipoproteins. The gene expression of apolipoprotein A and apolipoprotein B has been found to be regulated by zinc and copper (Zhang et al., 1995; Reaves et al., 2000; Cui et al., 2002) and more importantly, another AD-related apolipoprotein, clusterin (apolipoprotein J; Jones, 2010), is increased after zinc exposure (Trougakos et al., 2006). It is possible that altered metal levels in AD patients might affect the expression/transportation of apolipoproteins, including ApoE. Alternatively, metals might have different effects on the expression/transportation of the three ApoE isoforms, accounting for the different risk levels for AD among the three allele carriers.

Finally, studies have shown that ApoE can regulate synaptic zinc and glutamate levels in the hippocampus. The depletion of ApoE leads to a reduced expression of ZnT3, in parallel with a reduction in synaptic zinc content in APOE knockout mice, suggesting the ApoE modulates zinc homeostasis in the brain (Lee et al., 2010). The synaptic zinc is required for long-time potentiation (LTP) and is critical for the proper functioning of hippocampal circuitry in health and disease (Pan et al., 2011). So decreased ApoE levels would lead to synaptic zinc deficiency and cognitive impairments. Studies with human APOE Targeted Replacement (TR) mice demonstrated that compared to APOE2 and APOE3 TR mice, APOE4 TR mice have decreased levels of glutamate (Dumanis et al., 2013), which is an excitatory neurotransmitter co-released with zinc at the synapse during neuronal activity, and which is important to maintain normal hippocampal LTP and cognitive function (Paoletti et al., 2009). Thus, this may contribute to the increased risk of neurodegeneration associated with APOE4 carriers.

**Figure 1:**
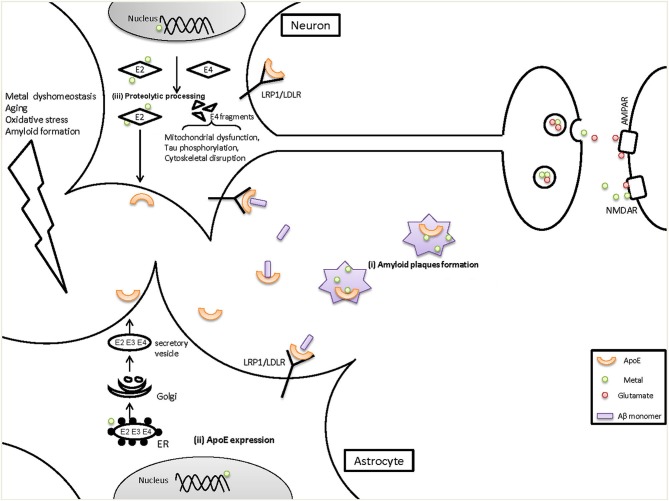
**The potential role of metals on ApoE in the pathogenesis of AD**. ApoE4 is a risk factor for the onset and development of AD, but the mechanisms are not fully understood. We have provided evidence for several points of interaction between metals and ApoE in AD, as shown here. (i) It has been demonstrated that both metals (zinc, copper and iron) and ApoE accumulate in the amyloid plaques, which could cause metal dyshomeostasis (less functional metals available) and decreased ApoE levels in the brain. (ii) Metal dyshomeostasis in AD patients might influence the expression of ApoE in astrocytes which are the main source of brain ApoE, resulting in decreased ApoE levels. ApoE levels can be affected by transcriptional level in nucleus and/or within rough endoplasmic reticulum (ER) where ApoE is synthesized. Reduced ApoE levels would contribute to AD pathogenesis as ApoE can mediate Aβ clearance through LRP1/LDLR and helps to maintain the vesicular zinc and glutamate levels at synapse. (iii) In response to aging, Oxidative stress and amyloid formation, neurons turn on or increase their expression of ApoE. However, neuron-ApoE is cleaved and generate C-terminal truncated fragments. ApoE4 is much more susceptible than ApoE2 and ApoE3. In the proteolysis of ApoE, metals bind to ApoE2 and stabilize its intact structure whereas ApoE4, which has a decreased affinity for metals, tends to be degraded to fragments. ApoE4 fragments can induce severe impairments to mitochondrial function and to the cytoskeleton, leading to neurodegeneration. Additionally, more ApoE2 is secreted in the brain compared with ApoE4, which will then impact various brain functions such as maintaining the normal levels of synaptic zinc and glutamate. In contrast, decreased levels of ApoE4 would reduce their levels and impair hippocampal LTP and then cause cognitive damage.

Taken together, these findings support an interaction between metals and ApoE that may be important in the pathogenesis of AD (Figure 1). In this review we summarized the evidence showing that metals bind to ApoE in an isoform-specific way, and that ApoE modulates metal homeostasis in the brain. There is also the possibility that metals may regulate ApoE levels. However, some key issues need to be directly addressed to provide definitive evidence for a metal:ApoE interaction, including the following: (1) Do metals (apart from zinc) have differential affinities for the three ApoE isoforms?; (2) Is the stability or degradation of ApoE isoforms affected by metal binding?; (3) Are changes in neuron- and astrocyte-specific ApoE expression/transportation caused by altered metal levels in AD? If so, what’s the underlying mechanism? Thus, further study is required for an integrated understanding of the interactions between metals and ApoE, and how they act together in the development and progression of AD.

## Conflict of interest statement

The authors declare that the research was conducted in the absence of any commercial or financial relationships that could be construed as a potential conflict of interest.
